# Designing mobile health to align with the social determinants of health

**DOI:** 10.3389/fdgth.2023.1193920

**Published:** 2023-05-18

**Authors:** Courtney C. Rogers, Sophia S. Jang, Whitney Tidwell, Sarah Shaughnessy, Juliane Milburn, Fern R. Hauck, Ishan C. Williams, Rupa S. Valdez

**Affiliations:** ^1^ Department of Systems and Information Engineering, University of Virginia, Charlottesville, VA, United States; ^2^ Department of Public Health Sciences, University of Virginia, Charlottesville, VA, United States; ^3^ Richmond City Health District, Richmond, VA, United States; ^4^ City of Raleigh Municipal Government, Raleigh, NC, United States; ^5^ Department of Family and Community Health Nursing, Virginia Commonwealth University, Richmond, VA, United States; ^6^ Department of Family Medicine, School of Medicine, University of Virginia, Charlottesville, VA, United States; ^7^ School of Nursing, University of Virginia, Charlottesville, VA, United States

**Keywords:** maternal health, health inequities, social determinants of health, health informatics, patient ergonomics

## Abstract

The maternal health crisis in the United States is becoming increasingly worse, with disparities continuing to escalate among marginalized populations. mHealth can contribute to addressing the Social Determinants of Health (SDOH) that produce inequities in maternal morbidity and mortality. Reducing inequities through mHealth can be achieved by designing these technologies to align with SDOH. As mHealth developed to support maternal health has primarily supported the extension of clinical care, there is an opportunity to integrate frameworks and methods from human factors/ergonomics and public health to produce thorough comprehension of SDOH through intentional partnerships with marginalized populations. Potential for this opportunity is presented through a case study derived from a community-based participatory research process focused on transportation access to maternal health services. Through multi-faceted, interdisciplinary, and community-based approaches to designing mHealth that attends to the systemic factors that generate and escalate inequities, improvements in the maternal health crisis could be realized.

## 1. Introduction

The maternal mortality rate in the United States continues to worsen in comparison to other high-income countries (1) and disparities among marginalized populations are widening (2). Structural and social factors have been found to considerably affect disparities in maternal mortality and morbidity (3, 4). These factors are rooted in systems of oppression, which have intentionally produced adverse social and economic conditions among marginalized populations. These conditions are referred to in the public health literature as the Social Determinants of Health (SDOH) (5). Multiple U.S. federal agencies and the World Health Organization have underscored the importance of addressing SDOH to improve health equity, including as related to maternal health (6–9).

Mobile health (mHealth) technologies have mostly been developed as low-cost interventions to extend clinical care into virtual environments and to promote disease self-management. Current technologies designed for maternal health focus primarily on providing clinical care such as telehealth, diagnostics, and remote monitoring (10). However, clinical care is only one contributing factor to health outcomes, as SDOH can be attributed to 50% of health outcomes (11). mHealth could contribute to improving communication across settings, access to a broad range of services, and continuity of care across health and human services (10). As such, mHealth designed to promote maternal health should extend beyond offering clinically-focused care and encompass considerations for SDOH, explicitly supporting birthing people who experience inequities produced by adverse SDOH. To extend the applications of mHealth designed to improve maternal health, there is a need to understand how technologies can be designed to align with SDOH.

Human factors/ergonomics (HF/E) can contribute to this imperative as frameworks such as the patient work system have previously been used as a basis to address health equity (12) and derive consumer health informatics design guidance (13). The patient work system framework, however, has a strong focus on studying patient work that arises from the medical management of an illness (e.g., medication management (14)). Though this framework captures the broader factors that shape patient work, there is an opportunity to explicitly examine the facets of patient work that arise from SDOH and how these interact within a patient work system. In this paper, we discuss how HF/E frameworks and methods should be merged with those of public health to inform the design of mHealth technologies that attend holistically to improving maternal health outcomes. We present a case study centering one determinant of health (i.e., transportation access), providing design opportunities for mHealth. Our perspectives draw from existing literature as well as our experiences as academic researchers, engineers, health care professionals, and public health professionals.

## 2. Extending the patient work system to encompass SDOH

Patient work is the “exertion of effort and investment of time on the part of patients or family members to produce or accomplish something” (15). Patient work takes multiple forms: illness work, everyday life work, biographical work, and articulation work (15). This work can be invisible (16), biform in nature (17), and also have collaborative elements (18). Patient work is shaped by a work system inclusive of the following contexts: person, task, tools/technologies, organizational, physical, and social (19). The study of patient work and the work system has often centered around illness work, for example, tasks like medication and symptom management (14, 19, 20), which take a traditionally narrow conceptualization of health. Yet, from the perspective of SDOH, which are “the conditions in the environments where people are born, live, learn, work, play, worship, and age (9),” health is more broadly conceptualized to include activities such as attending school, having a safe place and neighborhood to live in, and accessing reliable and affordable transportation, expanding to all types of work articulated above. The work system provides a perspective to study how components interact and how components in a system could be hindering or facilitating a broad range of patient work. By examining work that arises from SDOH and tying this explicitly into the patient work system, HF/E and public health perspectives can be merged to develop more comprehensive understandings of systems.

This holistic understanding can contribute to designing mHealth that better aligns with reality, which could improve the appropriateness, adoption, and effectiveness of these technologies. By extending the patient work system to encompass SDOH, aspects of the work that are unknown or undervalued by professionals (i.e., invisible work) and the product of their interactions (i.e., invisible work system can be elucidated (16). This extended model can be used as a basis to study how health-related work can be supported by aligning consumer and clinical mHealth technologies (13, 21) to support collaborative work (18) and collaborative health IT (22). Taken together, aligning theoretical concepts across disciplines produces more inclusive models of the systems in which mHealth is being designed and implemented. To develop these models, future inquiry should focus on the research questions presented in Box 1.

Box 1 **Future Research Directions to Extend the Patient Work System to Encompass SDOH and Inform mHealth Design.**

1. What sub-factors need to be added to the patient work system to encompass SDOH?
2. How can patient work arising from SDOH be identified and characterized in a patient work system?
3. In a patient work system that encompasses SDOH, how can interactions be identified and characterized?
  a. How can these interactions inform the design of mHealth that aligns with SDOH?
4. How can a patient work system that encompasses SDOH be used to identify components of invisible work and elucidate invisible work systems?
  a. How can the identification of components of invisible work and the elucidation of invisible work systems be translated into the design of mHealth that aligns with SDOH?
5. How can a patient work system that encompasses SDOH help identify components of collaborative work?
  a. How can the identification of components of collaborative work be translated into the design of mHealth that aligns with SDOH?

## 3. Merging participatory ergonomics and community-based participatory research

Designing mHealth to align with SDOH requires thorough comprehension of social, cultural, historical, and political contexts that have produced differential impacts on marginalized communities. From an HF/E perspective, participatory ergonomics was developed to include employees in an organizational environment in the design process (23, 24). This approach is just beginning to be expanded to working with historically marginalized populations within community contexts (25). Community-based participatory research (CBPR) is a public health methodology that has been widely employed in the discipline specifically for establishing research partnerships with marginalized communities. In this approach, researchers and community stakeholders engage as equal partners in all steps of the research process to collectively produce evidence for social change (26). Most importantly, this approach helps to establish trust, especially in marginalized communities that have been exploited by the scientific community. CBPR is grounded in the same principles as and shares similar processes to participatory ergonomics, including earlier stages of co-defining design problems to evaluating and implementing design guidance. Particular to mHealth development in community-based work, it is imperative to question whether a technological intervention is desired by the community (27). This requires a significant investment of time to fully comprehend the issues the community would like to address and the types of interventions they are interested in developing (28–30). As participatory ergonomics is newly being implemented in community contexts and CBPR has an established history in partnering with marginalized populations for public health research, there is potential to merge these methodologies to create interdisciplinary approaches for the purposes of addressing SDOH through digital health.

## 4. Case study: transportation access to maternal health services

**Figure 1:**
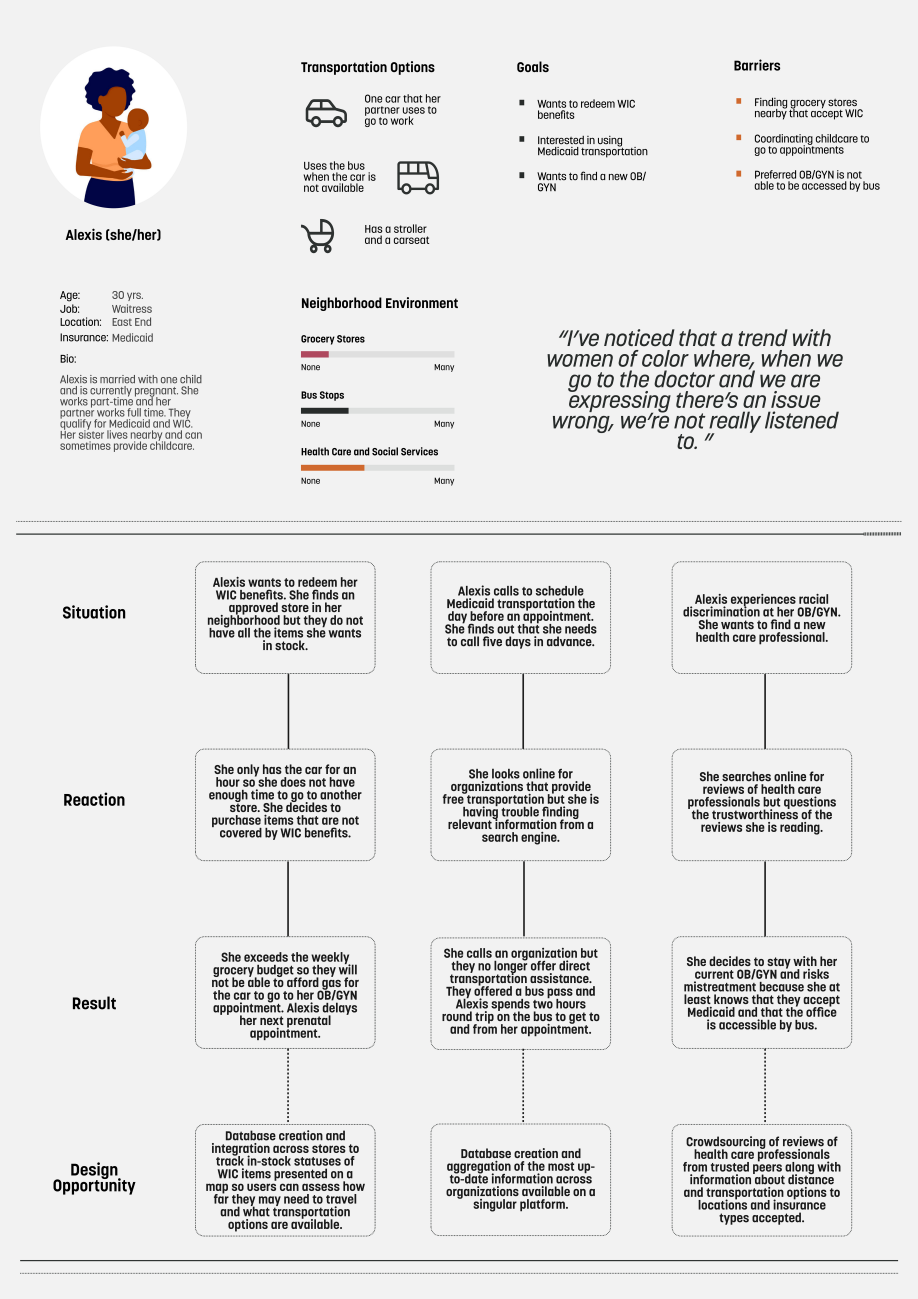
Persona, scenarios, and design opportunities.

As SDOH are broad and complex in nature, one way to begin to design mHealth to align with SDOH is to focus on how the components of the patient work system can be expanded by examining one determinant and how patient work stemming from that determinant interacts with other components across a system. Transportation access is one SDOH that affects prenatal care utilization, access to postpartum care, and the management of underlying chronic conditions during and after a pregnancy (31, 32). Structural discrimination embedded in the design of transportation systems has resulted in disproportionately low access to healthcare and public services in marginalized communities (33, 34). Currently, there is a paucity of mHealth technologies that incorporate considerations for transportation access as it relates to maternal health. The technologies available have a narrow focus on accessing clinical services by supporting the delivery of monetary resources to pay for transportation and direct coordination of transportation (35, 36). To expand on current technologies, Figure 1 presents a persona and scenarios grounded in qualitative interview data collected as part of a CBPR process focused on exploring transportation access to maternal health services through a patient work system lens (37). Scenarios are derived from the persona and are coupled with mHealth design recommendations to address the challenges posed in the scenarios. These recommendations serve as examples to illustrate how mHealth can be designed to align with SDOH and more research is needed to capture the breadth and depth of the lived experience of birthing persons with a diverse range of identities across a multitude of settings. It should be noted that these recommendations are not comprehensive and should be co-designed and co-developed with community members. This process could include approaches such as participatory design workshops (38). Methods such as affinity diagramming and dot voting can be used to thematically categorize and prioritize, respectively, design ideas (39). Moreover, additional determinants should be explored in-depth to create mHealth technologies that holistically attend to a wide range of SDOH.

## 5. Ethical considerations

Since marginalized populations are more likely to experience adverse SDOH, it is imperative to consider the ethical implications of mHealth designed to attend to SDOH, as there is a risk of erpetuatingexisting biases and inequities. As a result, mHealth interventions should be co-designed and co-evaluated with those experiencing inequities. Sustainability of these interventions must also be considered before engaging in the design process. Beyond the norms for protecting health-related information (40), those using these technologies should have agency over their data (29). This agency includes customizable features related to what types of data are shared and to whom as well as how this data can be used for research. Lastly, these technologies also need to be designed to be accessible for all disability types and responsive to intersectional identities (28, 41).

## 6. Conclusion

As a field focused on promoting human well-being in complex socio-technical systems, HF/E has frameworks and methods to contribute to improving maternal health by designing mHealth to align with SDOH. This extension of approaches will require expanding current understandings of patient work and patient work system as well as integrating participatory methodologies across disciplines. Designing mHealth to align with SDOH could contribute to dismantling the systemic factors that produce stark inequities in maternal mortality and morbidity among marginalized populations.

## Data Availability

The original contributions presented in the study are included in the article, further inquiries can be directed to the corresponding author.
